# Relearning and Retaining Personally-Relevant Words using Computer-Based Flashcard Software in Primary Progressive Aphasia

**DOI:** 10.3389/fnhum.2016.00561

**Published:** 2016-11-16

**Authors:** William S. Evans, Megan Quimby, Michael Walsh Dickey, Bradford C. Dickerson

**Affiliations:** ^1^Geriatric Research Education and Clinical Center, VA Healthcare SystemPittsburgh, PA, USA; ^2^Frontotemporal Disorders Unit, Department of Neurology, Massachusetts General HospitalBoston, MA, USA

**Keywords:** primary progressive aphasia, rehabilitation, computer-based treatment, distributed practice, aphasia, spaced retrieval

## Abstract

Although anomia treatments have often focused on training small sets of words in the hopes of promoting generalization to untrained items, an alternative is to directly train a larger set of words more efficiently. The current case study reports on a novel treatment for a patient with semantic variant Primary Progressive Aphasia (svPPA), in which the patient was taught to make and practice flashcards for personally-relevant words using an open-source computer program (Anki). Results show that the patient was able to relearn and retain a large subset of her studied words for up to 20 months, the full duration of the study period. At the end of treatment, she showed good retention for 139 words. While only a subset of the 591 studied overall, this is still far more words than is typically targeted in svPPA interventions. Furthermore, she showed evidence of generalization to perceptually distinct stimuli during confrontation naming and temporary gains in semantic fluency, suggesting limited gains in semantic knowledge as a result of training. This case represents a successful example of patient-centered treatment, where the patient used a computer-based intervention independently at home. It also illustrates how data captured from computer-based treatments during routine clinical care can provide valuable “practice-based evidence” for motivating further treatment research.

## Introduction

Primary progressive aphasia (PPA) is a neurodegenerative disorder affecting language in the relative absence of other deficits (Mesulam, 2013). It is organized into subtypes, with the semantic variant Primary Progressive Aphasia (svPPA) characterized by impaired naming, single-word comprehension, object knowledge and surface dyslexia, and relatively spared repetition, articulation and grammar (Gorno-Tempini et al., 2011). There is an ongoing need to develop more effective and resource-efficient treatments for this disorder.

Below, we present treatment outcomes for a patient with svPPA who received a novel naming treatment using an open-source computer-based flashcard program called Anki. Treatment took advantage of Anki’s adaptive distributed practice algorithm to enable the patient to efficiently relearn and retain a much larger number of personally-relevant words than in existing treatment approaches.

In this report, we first describe the patient’s presentation and changes over 2 years. We then briefly review relevant treatment literature (see “Overview of Naming Treatment in svPPA” Section), followed by rationale for the computer-based treatment (see “Development and Rationale of Current Treatment Approach” Section). We then present treatment procedures and results (see “Treatment Procedures” and “Treatment Results” Sections), followed by interpretation and conclusions (see “Treatment Discussion” and “Concluding Remarks” Sections).

The patient was a 72 year-old right-handed woman. She was referred to the Massachusetts General Hospital PPA program within the Frontotemporal Disorders Unit in fall 2013 for suspected PPA, with word-finding deficits and difficulty following complex conversation. She first noticed language difficulties in 2003 (at age 62), primarily in remembering proper nouns. However, her difficulties had slowly increased, with a marked increase in the 2–3 years preceding referral. She had a doctorate-level education and worked part-time at referral, although she stopped working soon afterwards. Prior medical history showed only hypertension and Graves’ disease. The patient had developed a detailed and well-organized compensatory notebook strategy, keeping lists of personally-relevant problematic words and their definitions, organized by category. She and her spouse reported that she spent significant time reviewing and adding to her notebooks, and that that she found them helpful for work and daily communication.

**Figure 1:**
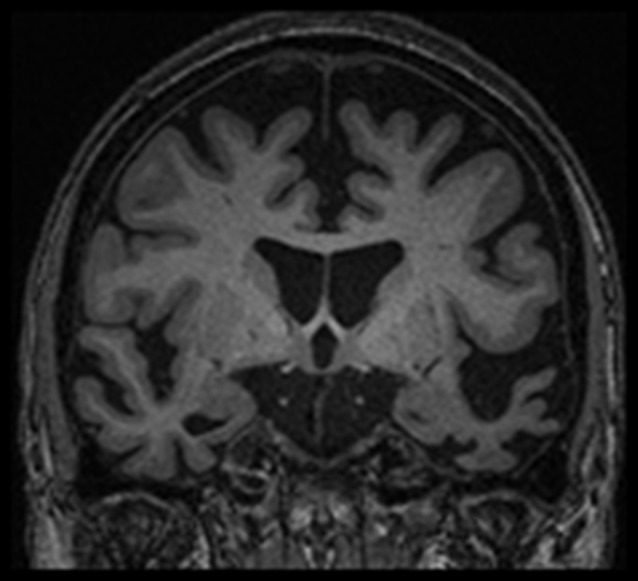
**Magnetic resonance imaging for the patient from fall 2013, showing predominantly left-hemispheric cortical atrophy, most prominent in the anterior temporal lobe.** Shown as per radiological convention (left side on right).

At referral, behavioral testing and imaging were most consistent with diagnosis of svPPA. [^18^F] Fluorodeoxyglucose Positron Emission Tomography revealed marked hypometabolism in the anterior temporal lobes, slightly more pronounced on the left than the right, and relatively mild hypometabolism in the left frontal lobe. Magnetic Resonance Imaging found predominantly left-hemispheric cortical atrophy, most prominent in the anterior temporal lobe (Figure 1).

Following referral, the patient was enrolled in a longitudinal research study with the second and fourth authors, which tracked her language performance over time. Written consent by the patient and her spouse was obtained under a protocol approved by the Partners Human Research Committee Institutional Review Board. Initial testing (Assessment 1, Table 1) revealed mild auditory and written comprehension deficits, mild-moderate semantic deficits, mild repetition deficits and severe confrontation-naming deficits. Over time, the patient’s performance declined across these areas, with notable exceptions in confrontation naming on the Boston Naming Test (BNT) and verbal semantic fluency. In these areas, the patient showed marked increase in performance during Assessment 2. The patient had been referred to the first author for outpatient language treatment following Assessment 1, and when the second author noted the patient’s subsequent testing gains, we began to discuss these large and unexpected treatment effects (see “Case Discussion” Section).

**Table 1:** **Patient language performance over time**.

	Assessment 1 (October 2013)	Assessment 2 (November 2014)	Assessment 3 (May 2015)	Assessment 4 (November 2015)
**WAB**
Auditory sequential commands	70/80	70/80	50/80	52/80
Repetition	90/100	87/100	87/100	84/100
Reading sentence comprehension	18/40	20/40	22/40	14/40
Reading commands: decoding	10/10	10/10	10/10	10/10
Reading commands: performance	10/10	9/10	8/10	8/10
**CSB: word-picture matching**
Living	27/32	16/32	16/32	10/32
Manmade	26/32	26/32	23/32	21/32
(Total)	(53/64)*	(42/64)*	(39/64)*	(31/64)*
**Verbal fluency (60 s)**
Letter fluency (F, A, S)	14	7*	6*	7*
Semantic fluency (Animals, Vegetables)	10	15	7*	3*
**BNT- Short form**	1/30*	15/30*	6/30*	5/30*

*Note: WAB, Western Aphasia Battery (Kertesz, 1982), CSB, Cambridge Semantic Battery (Bozeat et al., 2000), BNT, Boston Naming Test (Kaplan et al., 1983). This 30-item short form of the BNT was normed by Williams et al. (1989). Asterisks indicate scores below published cutoff values for neurologically healthy adults (more than 2 SDs below age- and education-adjusted means) on noted tests. Published cutoff values not available for WAB subtests*.

## Background

### Overview of Naming Treatment in svPPA

The majority of the svPPA treatment literature to date involves case studies targeting lexical retrieval. A recent meta-analysis (Jokel et al., 2014) reviewed at total of 39 studies involving behavioral naming treatment for 41 SD/svPPA patients. Across studies, Jokel et al. (2014) found that treatment led to improved naming in almost all cases for trained words, but that generalization to untrained items or tasks was severely limited (e.g., Graham et al., 1999; Dressel et al., 2010), with the naming of trained word-picture pairs only generalizing to perceptually similar pictures of the same items (e.g., Green Heredia et al., 2009; Jokel and Anderson, 2010). Partially-intact semantic knowledge also appeared to be helpful for long-term maintenance of treatment effects (Snowden and Neary, 2002). However, Jokel et al. (2014) claimed that in general, observed treatment gains appear to rely on episodic memory systems, due to limited treatment generalization. Abstracting from these studies, they developed a set of treatment recommendations for svPPA, which included involving patients in selecting personally relevant targets and using semantically-based training. They also recommended computer-based approaches and independent home practice as effective and cost-efficient treatment options.

### Development and Rationale of Current Treatment Approach

Existing svPPA naming-treatment studies have generally treated a relatively small number of words via intensive training (e.g., 30–40 words: Savage et al., 2013). However, efficient training more words directly could have a greater functional impact. Spaced repetition (e.g., Sohlberg et al., 2005) offers one promising means to accomplish this. In adaptive versions of this technique, a learning algorithm is used to adjust the review frequency, with high-accuracy stimuli practiced less frequently, and low-accuracy stimuli practiced more frequently. Adjusting the repetition spacing for each item in this way allows for maximum learning and retention in limited study time (Woźniak and Gorzelańczyk, 1994). There is some evidence supporting the use of spaced retrieval for naming in post-stroke aphasia (Fridriksson et al., 2005) and dementia (Brush and Camp, 1998), and in one case with semantic dementia (Bier et al., 2009). Motivated by this background, a novel treatment using a computer-based flashcard program was developed, building on the patient’s good cognitive functioning, strong motivation and existing compensatory notebook strategy. The main goals of the treatment were to: (a) efficiently and directly train naming for “lost” words using spaced retrieval and personal episodic memory associations; (b) strengthen and maintain partially-degraded semantic representations via semantic feature generation; and (c) maximize patient independence and self-efficacy via personally-relevant stimuli selection and clinician-supported home computer-based practice.

## Case Discussion

### Treatment Procedures

The computer-based treatment designed for this patient used the open-source software program Anki^1^. This free, cross-platform program supports multimedia flashcards including pictures, text, sound recordings and video. Flashcards are reviewed using an adaptive scheduling algorithm, with review frequency based on self-rated accuracy. Anki flashcards contained pictures and written descriptions as prompts. The written target word was the answer. For each card, the practice routine was as follows:

1. The picture and description prompt appeared, and the patient attempted to name the target.
2. Instead of guessing, if she could not spontaneously name the target, she clicked “show answer” and read aloud the correct response. This was intended to avoid encoding of error responses, consistent with errorless learning techniques (Sohlberg et al., 2005).
3. Whether or not she could name the target, she then attempted to generate three features from using a list of cloze sentence cues for personally-relevant episodic (“[Target word] reminds me of [‥.]” or semantic information “a [Tuba] is a type of [musical instrument]”). She repeated the target word in each cloze sentence, which was intended to strengthen associations between the target and activated semantic/episodic information.

The patient was taught to follow the above practice protocol and make flashcards for personally-relevant words, using the internet to acquire pictures and descriptions. Treatment consisted of 24 1-h outpatient sessions over 20 months. The first 10 sessions occurred once weekly until the patient could complete all flashcard-generation and practice procedures independently. During the training process, a written handout describing card creation and practice was provided (see “Supplementary Materials”).

### Treatment Results

Data analysis and visualization were conducted in RStudio (version 0.98.1028). Practice data were extracted from Anki, consisting of 10,582 trials (130 h of practice) over 20 months of treatment. During this period, the patient studied at home approximately 30 min per day, 3–4 times per week. She reviewed approximately 35 flashcards per session and made a total of 591 unique flashcards.

The primary research question was whether the patient was able to retain and relearn the words she practiced using Anki. Anki’s built-in scheduling provided a natural way to address these questions, via the flashcards’ *study interval* ^2^. This variable reflects how many days a given flashcard is predicted to be retained in memory without additional review. Study interval updates in nonlinear fashion after each practice trial, increasing following successful trials and decreasing following unsuccessful trials. For this patient, flashcards in the initial “learning phase” received reviews at 1-min and then 10-min intervals until they were consistently answered correctly. The study interval for successfully “learned” flashcards was set to vary between 1 day and 28 days. Thus, a flashcard with a high study interval (close to 28 days) was one that was well learned and retained (having been answered correctly multiple times in a row at increasing time intervals), while a flashcard with a low study interval (at or near 1 day) was one poorly retained, with frequent incorrect responses.

To investigate whether the patient retained words she practiced using Anki, we first examined flashcard study interval. At the end of the 20 months, the mean final flashcard study interval was 4.59 days, significantly greater than the learned flashcard minimum of 1 day (*t* = 9.96, *df* = 590, *p* < 0.001). This indicates that the patient used Anki to successfully retain trained word-picture pairings. However, this overall effect was driven by a relatively small subset of her 591 flashcards: only 139 had study intervals greater than 1 day. For this “well-learned” subset, mean final study interval was 18.99 days.

**Figure 2:**
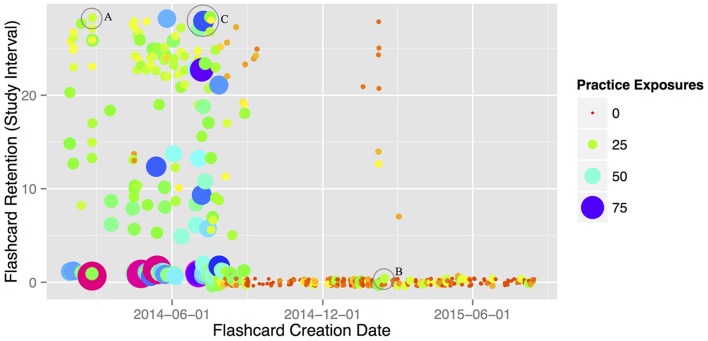
**Flashcard memory retention (as measured by Anki study interval), plotted by creation date and number of practice exposures for each created flashcard.** Two of her 591 cards (“cardinal” and “cockroach”) had over 100 exposures each, and were removed here for plotting purposes (but not from the corresponding analyses). Circled datapoints A, B and C represent specific practiced items; see text in “Treatment Results” above for discussion.

To further investigate flashcard retention, we examined the effects of *practice exposure* (how many times each flashcard was reviewed) and *practice start date* (the date each was added). Figure 2 shows that most well-retained flashcards were introduced during the first 10 months of treatment. However, it was unclear whether this was due to the patient’s declining language ability or reduced practice exposure, as older flashcards also had more practice exposures. Therefore, practice start date was split into two treatment phases (first vs. second half) and entered along with practice exposures into a multiple linear regression predicting final flashcard study interval. Effects of exposure and start date were both significant (practice exposures: *β* = −0.04, *t* = −2.07, *p* = 0.04; practice start date: *β* = −9.68, *t* = −12.40, *p* < 0.001): retention for words added in the first half of treatment was significantly greater than for words from the second half, even when controlling for practice exposure differences. This effect is best illustrated by comparing example flashcards “A” (“doornob”) and “B” (“sofa”) in Figure 2; although both of these flashcards received 21 practice exposures, only flashcard A was well-retained (had a high final study interval). This suggests that practicing earlier in the disease process may have protective effects. However, aspects of the patient’s Anki treatment were also changing over time (e.g., the total number of studied flashcards). This may also have affected her memory retention.

To investigate whether the patient was able to relearn words she practiced using Anki, we next examined final retention of flashcards that “lapsed” at least once during the treatment period. Lapsed flashcards are ones that are answered incorrectly after having been successfully learned. If the patient was *unable* to relearn flashcards once she began to forget them, this would appear as low final study intervals (i.e., close to 1 day) for all flashcards classified as lapsed. However, the mean final study interval for “lapsed” flashcards was 9.4 days overall, significantly greater than the 1-day minimum (*t* = 11.15, *df* = 159, *p* < 0.001). This indicates that the patient was able to relearn at least some forgotten words after additional Anki practice. This finding is best illustrated by comparing flashcards “A” and “C” (“esophagus”) in Figure 2: although flashcard A never lapsed during the treatment period, flashcard C lapsed three times (hence the high number of repetitions, since it went back through the learning phase multiple times). However, both A and C were equally well-retained at the end of treatment.

As discussed in the “Introduction” Section, we first became interested in this patient’s case when the second author noted a marked increase in her BNT naming performance during Assessment 2 (from 1/30 to 15/30; Table 1). While investigating this unexpected improvement, we discovered the patient had added BNT words to Anki between Assessments 1 and 2. After discussing this with the patient and her spouse, we determined that the patient had likely written down BNT words in one of her notebooks following Assessment 1, then added them to Anki while looking through her notebooks for functional targets.

**Figure 3:**
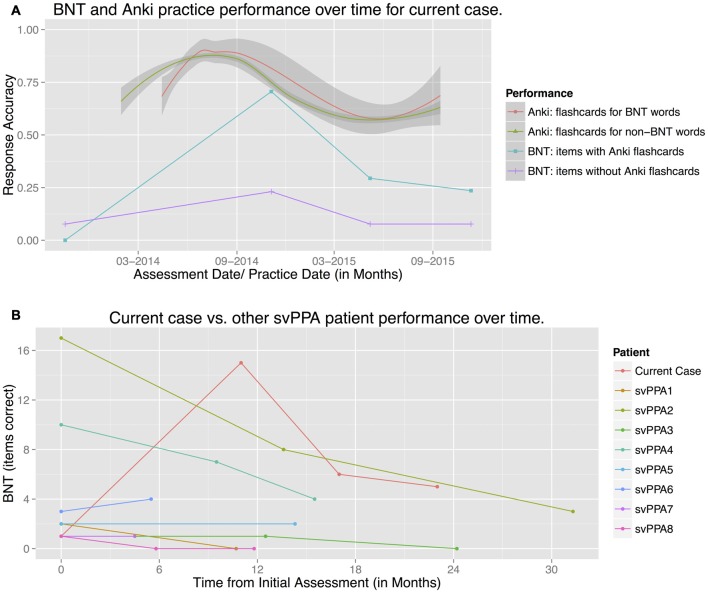
**Patient Boston Naming Test (BNT) performance over time, compared to (A)** Anki practice performance and **(B)** other svPPA patient BNT performance. **(A)** The two lower linear plots show the patient’s standard BNT performance at Assessments 1–4 (as listed in Table 1), plotted separately for words with Anki flashcards (*N* = 17, blue, squares), and for words without flashcards (*N* = 13, purple, crosses). The two upper nonlinear plots reflect smoothed accuracy performance (loess lines) for Anki practice over the 20-month treatment period, separately for flashcards depicting BNT words (*N* = 17, orange, circle), and all remaining non-BNT flashcards (*n* = 574, green, triangle). The patient used Anki for approximately 3 months before adding any flashcards for BNT words, hence the difference in starting points. **(B)** BNT performance (total items correct), plotted over time for the current case (orange) and a comparison group of other svPPA patients being tracked longitudinally (*N* = 8).

This negatively affected the validity of the patient’s BNT test results, and while certainly patient-centered, this highlights a potential pitfall of unsupervised target selection. However, she only made flashcards for approximately half of the BNT (*N* = 17), providing a useful “natural experiment” for treatment generalization by allowing comparison of “Anki-trained” to “untrained” BNT words (Figure 3A). The bottom half of Figure 3A plots BNT accuracy performance across Assessments 1–4, for trained and untrained words (blue squares and purple crosses, respectively). The 20 months of available Anki practice data are plotted in the top half of the figure as loess-smoothed regression lines of trial-by-trial practice accuracy, for Anki BNT flashcards (*N* = 17, orange) and all other Anki flashcards (*n* = 574, green).

Figure 3A shows that the patient’s naming performance on the BNT was better for Anki-trained than untrained words. A repeated-measures logistic regression model of BNT test performance found a significant effect of Anki practice (*β* = 1.72, *z* = 2.03, *p* = 0.04), such that the patient was 5.58 times more likely to correctly answer Anki-trained vs. untrained BNT words.

Figure 3A also shows that Anki practice and BNT test performance showed roughly matching accuracy trends over time. A repeated-measures logistic regression model tested the relationship between BNT performance and Anki flashcard retention, excluding Assessment 1 (before Anki treatment began). In the model, Anki study interval (a measure of Anki memory retention for each word) was used as a continuous predictor of BNT naming performance for each Anki-trained word. The model showed a significant relationship between Anki retention and BNT training (*β* = 0.076, *z* = 2.43, *p* = 0.02): thus, better retention in Anki was directly related to better naming performance on the BNT. The low BNT performance at Assessment 1, before Anki treatment, suggests this relationship is causal. Since the flashcards used picture stimuli perceptually quite distinct from the line drawings on the BNT, we interpret this as evidence of generalization for trained Anki words.

As noted in the “Introduction” Section, this pattern of BNT performance is atypical. Figure 3B shows the patient’s BNT performance plotted against that of eight other svPPA patients enrolled in the same longitudinal study and meeting the same diagnostic criteria. While all other svPPA patients showed essentially flat or declining BNT performance over time, the current case’s performance improved and stayed above initial baseline. Mixed-effect Poisson regression showed that the slope of the current case’s performance over time differed significantly from the comparison group’s (*β* = 0.073, *z* = 2.62, *p* = 0.008)^3^.

### Treatment Discussion

Overall, results show that the patient was able to successfully retain and relearn personally meaningful words via self-generated flashcards and independent practice over a 20-month period. Although she was unable to successfully retain all 591 treated words by the end of this period, she did show good retention for 139 words, which is much larger than the ≤40 typically targeted in existing treatment approaches. The fact that her best-retained words were ones she began practicing earlier suggests that early review may have protective effects at later stages of disease progression. In addition, analysis of her “lapsed” words suggests that this approach supports relearning, and not merely retention.

The patient made Anki flashcards for approximately half of the 30 items on BNT short form. Subsequently, her naming improved significantly for these “trained” words compared to untrained BNT words. There was also a significant relationship between Anki memory retention and BNT naming performance, lesser improvement on untrained Anki words, and concomitant increases in semantic fluency, all which dropped off by the next testing point, six months later. These increases were in contrast to the more typical patterns of steady decline noted in eight other svPPA patients also being tracked longitudinally. When the patient made flashcards for BNT words, she used photographs (see Supplementary Figure S1 for a representative picture) that were perceptually quite distinct from the BNT line drawings. Previous work showing generalization of naming treatment generally only shows generalization between different exemplars when the two are very perceptually similar (Jokel et al., 2007; Green Heredia et al., 2009; Jokel and Anderson, 2010). However, generalization between more distinct exemplars has been shown in recognition memory tasks in SD when semantic knowledge for an item’s category is preserved (Graham et al., 2001).

Although generalization of naming from one distinct exemplar to another reflects only modest functional gains, it does represent an abstraction and generalization of learning that is semantic in nature. Evidence for generalization also appeared in semantic fluency and untrained BNT items, but not on letter fluency or word-picture matching. This suggests that Anki training temporarily increased activation of word names (and related semantic information such as category membership) for the purposes of production. However, this training did not improve semantic selection in the face of competitors, as would have been required to improve patient performance on the CSB word-picture matching (in this task, 10 targets from a single semantic category appear on each page).

If the patient’s testing gains reflect improved semantic processing based on relearned semantic knowledge, what systems were involved in this improvement, and why was it so transient? One possible explanation is that while most of her new learning relied on her relatively intact episodic system, generalization of this learning depended crucially on the involvement of residual semantic memory systems (Graham et al., 2001). As her semantic system continued to atrophy in the second year of the study (as seen on testing and per patient and family report), retention and some new episodic learning was still possible within the context of Anki, but generalization was not. If the generalization observed here reflects learning that, as we claim, is qualitatively semantic in nature, this would suggest that new semantic learning can be largely supported by nonspecialized hippocampal systems, but only in the context of some residual neocortical involvement (McClelland et al., 1995; Graham et al., 1999). This claim is consistent with previous observations that treatment effects rely primarily on intact episodic memory systems (Jokel et al., 2014), and also with observations that treatment and maintenance effects are largest when patients still possess partially-intact semantic memory abilities during training (Snowden and Neary, 2002; Jokel et al., 2006).

Although the outcomes of this intervention are promising, there are limitations to this case report. First, flashcards were self-selected and made by the patient. While this maximized the treatment’s patient-centered nature, this meant that pre-treatment baseline performance was not established. This should be addressed in future research. Second, creating flashcards for BNT words provided an opportunistic measure of treatment generalization, but future research should employ more rigorous, planned measures. Third, while the treatment showed some transient generalization effects to non-trained pictures and semantic fluency, no data are available regarding the effects of treatment in more functional contexts such as daily communication. It is likely that such effects were more limited. Finally, the patient only relearned and retained 139 of her 591 targeted words, most of which were added early in treatment. Therefore, issues of optimal flashcard set size and practice timing should be further explored.

## Concluding Remarks

Overall, this treatment represents a promising and practical approach to naming therapy in svPPA. It was feasibly implemented by a speech-language pathologist in an outpatient hospital clinic and appeared cost-effective: 24 total hours of billable direct patient contact over a period of 20 months resulted in 130 h of individualized drill-based home treatment, which the patient pursued with great perseverance and personal agency. Both the patient and her family reported that she found her home practice to be engaging and generally enjoyable, and stated it felt important that she had a way to attempt to improve her semantic and word-finding deficits directly, in addition to pursuing compensatory approaches and strategies. Given the promising findings in this case, follow-up research is warranted.

From a clinical perspective, one benefit of the current approach is that it used open-source software, freely available to both clinicians and patients. Although a single case report provides a low level of evidence (Yorkston et al., 2001), clinicians interested in applying this approach with appropriate clients should review the “Supplementary Materials”, which provide the training and treatment instructions used for this case.

While results are preliminary, this case report highlights exciting new rehabilitation options that are increasingly available via computer-based treatment. When appropriately designed, such approaches can improve the scope and cost-efficiency of rehabilitation services, while simultaneously maximizing patient locus-of-control.

Finally, this case is an example of how data captured in computer-based treatments can provide powerful “practice-based evidence” (Margison et al., 2000) during the course of routine clinical care, as case conclusions were drawn based on statistical analysis of 10,000+ practice trials from a 20 month period. Moving forward, such rehabilitation data sources will provide a powerful compliment to traditional group-based clinical treatment research, especially when integrated with electronic medical-records data.

## Author Contributions

Data collection, analysis and background literature review were conducted by WSE and MQ. Data interpretation and manuscript preparation were conducted by WSE, MQ, MWD and BCD.

## Funding

This research was supported by VA Rehabilitation Research and Development grant I01 RX000832 (MWD), National Institutes of Health grant R21 NS077059 (BCD), and the VA Pittsburgh Healthcare System Geriatric Research Education and Clinical Center (WSE and MWD). The contents of this article do not represent the views of the Department of Veterans Affairs or the United States Government.

## Conflict of Interest Statement

Although we do not believe it has affected the preparation of this work, we wish to note that BCD currently serves as a Frontiers review editor. The other authors declare that the research was conducted in the absence of any commercial or financial relationships that could be construed as a potential conflict of interest.

## Footnotes

^1^http://ankisrs.net/

^2^Details regarding Anki’s practice algorithm and setting options are available in the Anki manual: http://www.ankisrs.net/docs/manual.html

^3^This analysis is an extension of the case comparison approach suggested by Huber et al. (2015) using linear mixed-effect models.
