# Supplementary material for: Relearning and Retaining Personally-Relevant Words using Computer-Based Flashcard Software in Primary Progressive Aphasia
Source: Front Hum Neurosci. 2016 Nov 16;10:561. doi: 10.3389/fnhum.2016.00561 (PMC5110537; doi:10.3389/fnhum.2016.00561)
Supplement: Supplementary file 1 [file DataSheet_1.pdf]

frontiers

Supplementary Material

Relearning and Retaining Personally-Relevant Words using Computer-Based Flashcard Software in Primary Progressive Aphasia.

William S. Evans[1*], Megan Quimby[2], Michael Walsh Dickey[1], Bradford C. Dickerson[2].

[1]Geriatric Research Education and Clinical Center, VA Healthcare System, Pittsburgh, PA, USA

[2]Frontotemporal Disorders Unit, Department of Neurology, Massachusetts General Hospital, Boston, MA, USA.

- **Correspondence:**
  William S. Evans
  william.evans5@va.gov

1 Supplementary Data: Anki instructions provided to the patient

What follows are an edited version of the final written Anki flashcard creation and practice instructions provided to the patient during treatment. Readers interested in additional information related to using and installing Anki should refer to the Anki website (http://ankisrs.net/) and Anki user manual (http://www.ankisrs.net/docs/manual.html).

ANKI INSTRUCTIONS

What is the purpose of using Anki?
There are two main goals:

a. To give you a chance to practice and retain words that are important to you.
b. To give you a way to practice **describing the meaning of words** that will help you communicate better during conversations (even when you cannot remember the word you are looking for).

YOUR CURRENT PRACTICE ROUTINE:

Practice both your Word Practice deck using the “study now” option up to 30 minutes a day. Once you have put in your practice time, you can add new cards, edit ones you have made, or do internet research about the words you practiced today.

Do not spend too much time on Anki: aim for anywhere between 30 minutes and 1.5 hours per day for **all** your practice, browsing, and adding cards combined.

INFORMATION RELATED TO ADDING AND EDITING CARDS.

1. How do I look at my cards in a table format?
Click on "browse", then select "current deck" to see each of the cards listed.
To preview a card, select it in the list, then click the "preview" button on the top right of the page.

2. How do I add new cards to a deck?
First, select "word practice".
Then, click "add" at the top of the screen.
Now you should see the window for making cards.

3. How do I add information to cards?
On the "front", write in your description and definition.
Add the written target word to the "back".

Adding a picture:
Add a picture to the front by going to Google, typing in the word, then selecting the "images" option. Go through and pick an image that you like, click on it with two fingers, then pick the "copy image" option from the menu. Then, go back to Anki, click on the front of your card after the text. Hit the "enter/return" key, then paste in the picture below the text pressing "Command" + "V". (this means holding down the Command key, then tapping the V key once quickly). If you put in too many pictures, you can delete them with the cursor and the delete key.

Tags:
To put the word in a category (as you put it, "add a title"), choose category words to put in the "Tags" section (for example, "Animals", "Fruit", "Politics", etc.). If your category has more than one word in it, use "_" (for example, "Foreign_government").
It is ok to add more than one tag to each card! Please try to add 2 category tags to each card if you can think of them.

4. **What words should I pick for my cards?**
Choose words that you have a hard time remembering if…

a. you use the word a lot (for example, something you use every day to prepare food).
b. the word is related to something you care a lot about (for example, classical music).

5. How do I remove a card I do not want to practice anymore?
Go to "browse" for the deck it is in, and select the card by clicking on it in the list. Then, click the "suspend" button (it is in a menu above it, between "mark" and "change deck"). This saves the card in case you want it later, but does not put it into your practice anymore.

INFORMATION RELATED TO PRACTICING CARDS.

First, it is important to practice your cards regularly. 30 minutes a day, 4 days a week is your current recommendation. This is separate from time you spend adding new cards or editing ones you have already made.

1. How do I practice my cards?
When you open Anki, first click on you "word practice" deck.
Then, click "study now". Don't use the "browse" option to practice.

You want to practice each word by **making deep meaningful connections**, and by connecting saying the word with your description of it:

For each new card during practice:
First, **try to name the word**. If you can, then practice connecting **your descriptions to the word** by putting them together in a sentence. Make sure to say the word each time in the sentence to keep the word and its meaning connected. Use at least **three sentences** from the list below, or new sentences you come up with:

- "[ ] reminds me of..."
- "[ ] is/are..."
- "[ ] is/are related to..."
- "[ ] is/are a type of..."
- "[ ] is/are used for..."
- "[ ] look/looks like..."
- "[ ] have/has..."
- "[ ] might be found..."
- "[ ] is *not*..."
- "an example of [ ] is..."
  (Also, try to come up with **new** sentences like these if you can.)

For example, "Pigs are a type of animal used for food. Pigs usually have pink skin with floppy ears. Pigs remind me of the time I saw a one walking down the street in the city."

These sentences are the most important part of the practice, because they keep your brain active and the words connected to what you know about them.

If you can't remember the word after trying for 5-10 seconds, click "**show answer**", then practice the word in sentences just like above.

If you knew the word **without** needing to check the answer, click "good" to get the next card.
If you needed the answer to say or describe the word, click "again" instead.

2 Supplementary Figures

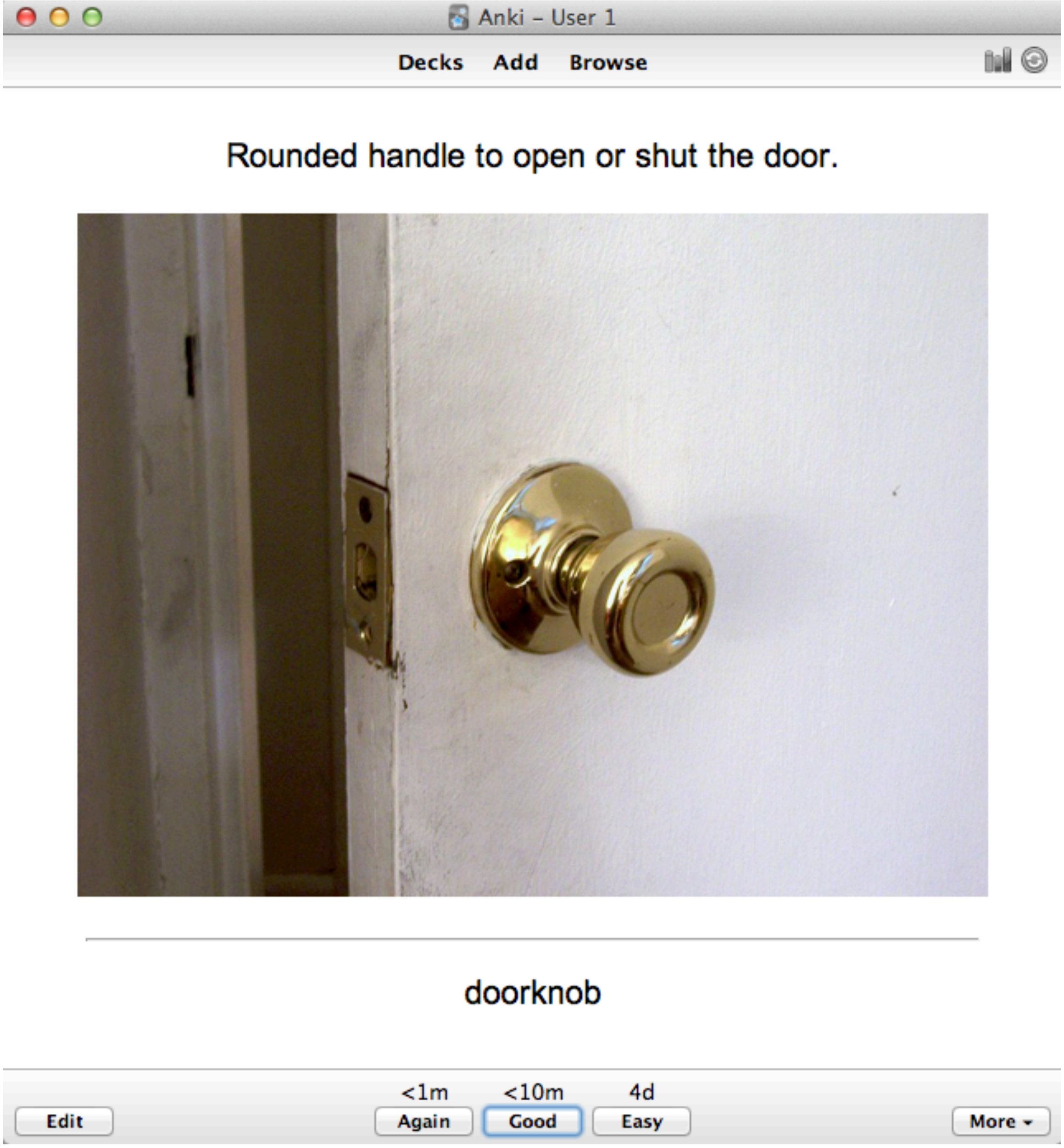

Supplementary Figure 1. Screenshot of a sample flashcard used in the patient's Anki treatment, depicting the "show answer" screen during the initial learning stage of practice. Each button at the bottom reflects a potential response that will update the flashcard's study interval a specific amount. "Again" (for an incorrect response), means the card will be scheduled for review in less than a minute, "Good" (for a correct response), in less than 10 minutes, and "Easy", in 4 days. Successive correct responses will continue to increase this interval, up to a maximum of 28 days for this patient. The text on this sample flashcard was written by the patient, but the picture she chose is replaced here for copyright purposes (current image released under Creative Commons).
